# Supplementary material for: The Eco-Bio-Social Factors That Modulate Aedes aegypti Abundance in South Texas Border Communities
Source: Insects. 2021 Feb 21;12(2):183. doi: 10.3390/insects12020183 (PMC7926310; doi:10.3390/insects12020183)
Supplement: Supplementary file 1 [file insects-12-00183-s001.zip › Supplemental/Materials S1-Surveys.docx]

**Materials S1:**

**Surveys-Knowledge Attitudes and Practices**

1. Do you agree to participate in this survey?

| Yes |  | No |  | **(Thank respondent and end survey)** |
| --- | --- | --- | --- | --- |

1. Gender of the family member surveyed (Observe. Don’t ask)

| Male |  | Female |  | Other/Not sure |  |
| --- | --- | --- | --- | --- | --- |

| **General Information of the Household** | | | | | | | | | | | | | | | | |
| --- | --- | --- | --- | --- | --- | --- | --- | --- | --- | --- | --- | --- | --- | --- | --- | --- |
|  | How long have you lived in the Rio Grande Valley Region?  ***(Read options)*** | | | | ☐< 1year  ☐1 year – 5 years  ☐5+ years | | | | | | | | | | ☐Lifelong resident  ☐Declined | |
|  | In the last 12 months has anyone in your household traveled outside the United States? | | | | ☐No  ☐ Yes 🡪 If yes, where? (List all countries) _____________________________________________________  ☐Not sure  ☐ *Declined* | | | | | | | | | | | |
| **Knowledge and Attitudes towards the vector and disease: We are going to ask you a few questions about mosquitoes and the diseases they transmit** | | | | | | | | | | | | | | | | |
|  | Do you think mosquitoes have an impact on you or your family? | | | | | | | | | | ☐ No **(SKIP TO 8)**  ☐ Yes  ☐ *Declined* **(SKIP TO 8)** | | | | | |
|  | What type of impact do you think they might have? | | | | ☐Health risk  ☐Nuisance  ☐Other (fill in) _________________________________________  ☐Don’t know/unsure | | | | | | | | | | | |
|  | Have you ever seen one of these?  ***(show flyer of pupae and larvae)*** | | | | ☐ No  ☐ Yes  ☐ *Declined* **(SKIP TO 9)** | | | | | | | | | | | |
|  | Do you know what these are? | | | | Write everything the interviewee says:  _____________________________________________________ | | | | | | | | | | | |
|  | Do you know where we might find them? | | | | Write everything the interviewee says:  _____________________________________________________ | | | | | | | | | | | |
|  | Have you ever seen these?  ***(show flyer of mosquitoes)*** | | | | ☐ No  ☐ Yes  ☐ *Declined* **(SKIP TO 19)** | | | | | | | | | | | |
|  | Do you know what these are? | | | | Write everything the interviewee says:  _____________________________________________________ | | | | | | | | | | | |
| **This type of mosquito is called *Aedes aegypti* and it transmits different type of diseases to people.** | | | | | | | | | | | | | | | | |
|  | When was the last time you saw a mosquito?  **Doesn’t need to be *Aedes***  ***(write the NUMBER in the box)*** | | | | ☐Never **(SKIP TO 19)**  ☐Days  ☐Weeks  ☐Months  ☐Years  ☐Don’t know | | | | | | | | | | | |
|  | Which time of the year would you say mosquitoes bother you the most? | | | | ☐Summer  ☐Winter  ☐Fall  ☐Spring | | | | | | | | | | ☐All year  ☐Never **(SKIP TO 19)**  ☐*NA* | |
|  | At what time of day would you say that mosquitoes bother you the most?  ***(select the options mentioned)*** | | | | | | | | | ☐At night (9:00pm to 3:00 am)  ☐During dawn (4:00 am to 6:00 am)  ☐In the morning (7:00 am to 10:00 am)  ☐Mid-day/noon (11:00 am to 1:00 pm)  ☐Afternoon (2:00 pm to 5:00 pm)  ☐Evening (6:00 pm to 8:00 pm  ☐NA | | | | | | |
|  | Have you been bitten in the past week inside your home? | | | | | | | | | ☐ No  ☐ Yes  ☐ *Declined* | | | | | | |
|  | Have you been bitten in the past week outside your home? | | | | | | | | | ☐ No  ☐ Yes  ☐ *Declined* | | | | | | |
|  | **Prompt if 15 or 16 is yes.** How many mosquito bites do you currently have? | | | | | | | | | ____________ | | | | | | |
|  | Are there specific areas nearby that you think are big sources of mosquitoes? | | | | Write everything the interviewer says:  _____________________________________________________ | | | | | | | | | | | |
|  | Currently, how many mosquitoes do you notice when you are INDOORS in your neighborhood? | | | | | | | | | ☐None  ☐Very few  ☐Moderate amount  ☐Quite a few  ☐Very many  ☐NA | | | | | | |
|  | Currently, how many mosquitoes do you notice when you are OUTDOORS in your neighborhood? | | | | | | | | | ☐None  ☐Very few  ☐Moderate amount  ☐Quite a few  ☐Very many  ☐NA | | | | | | |
|  | If you noted you had a mosquito problem on your property, what would be your first step to reduce the mosquitoes?  ***(Don’t read; check all that are mentioned.)*** | | | | | | ☐Call mosquito control  ☐Call the city or county  ☐Dump out the water  ☐Spray/treat themselves  ☐Hire mosquito control | | | | | | | | ☐Nothing; don’t care  ☐Other (fill in): _________________________  _________________________  ☐Don’t know/unsure  ☐ *Declined* | |
|  | How much of a problem do you think mosquitoes are in your community on a scale of 1 to 5 with 1 being not at all serious and 5 being extremely serious? | | | | | | | | ☐ 1. Not a problem  ☐ 2. Small problem  ☐ 3. Moderate problem  ☐ 4. Big problem  ☐ 5. Very big problem  ☐NA | | | | | | | |
| **The disease** | | | | | | | | | | | | | | | | |
|  | Do you know of any diseases that are transmitted by mosquitoes in the Rio Grande Valley?  ***(mark ALL that apply, “DO NOT” read)*** | | | | | | | | ☐Zika **SKIP TO 26**  ☐Chikungunya  ☐Dengue  ☐West Nile  ☐Malaria  ☐Other ____________________________________  ☐NA | | | | | | | |
|  | Before this interview, had you heard of the disease called Zika? | | | | | | | | ☐ No  ☐ Yes  ☐ *Declined* | | | | | | | |
|  | How serious a problem is Zika for the Rio Grande Valley on a scale of 1 to 5 with 1 being not at all serious and 5 being extremely serious?  **(Read the options if clarification is needed)** | | | | | | | | ☐ 1. Not at all serious  ☐ 2. Slightly serious  ☐ 3. Somewhat serious  ☐ 4. Very serious  ☐ 5: Extremely serious  ☐ *Declined* | | | | | | | |
|  | Where have you heard about Zika?  ***(mark ALL that apply, DO NOT read)*** | | **26.1 Person**  ☐Friend  ☐Health professional  ☐Family  ☐Teacher  ☐Neighbor  ☐Other ___________________ | | | | | | | | | | **26.1 Communication media**  ☐Television  ☐Radio  ☐Newspaper  ☐Internet  ☐Flyer  ☐Other _________________ | | | |
|  | Besides mosquito bites, do you know of any other ways that someone can get infected with Zika? | | | | | | | | ☐ Contact with sick people  ☐ Blood transfussion  ☐ Sexual intercourse  ☐ Other ___________________  ☐ NA | | | | | | | |
|  | From what you have heard what happens when someone gets infected with ZIKA?  ***(mark ALL that apply)*** | | ☐Fever  ☐Diarrhea  ☐ Miscarriage/Abortions  ☐ Microcephaly in babies  ☐ Nervous system damage in new born | | | | | | | | | | ☐ Joint pain  ☐ Rash  ☐ Headaches  ☐ Get sick till they die  ☐ Other _________________  ☐ NA | | | |
|  | Have you known anyone personally who has had dengue, chikungunya, or Zika? | | | | | | | | ☐ No  ☐ Yes  ☐ Not Sure  ☐ *Declined* | | | | | | | |
|  | If “Yes” prompt:  **In the Rio Grande Valley? Or other country?** | | Write everything the interviewee says:  _____________________________________________________ | | | | | | | | | | | | | |
|  | Is there something that worries you about Zika in particular? | | Write everything the interviewee says:  _____________________________________________________ | | | | | | | | | | | | | |
|  | Which means of communication would you say are the best to reach the members of your community, to talk about this disease?  ***(READ, mark ALL that apply)*** | | ☐Flyers  ☐Radio  ☐ Community gatherings  ☐ Television | | | | | | | | | | ☐ Internet  ☐ Social media  ☐ Other _________________  ☐ NA | | | |
| **Practices: Now I’m going to ask you a few questions about your home** | | | | | | | | | | | | | | | | |
|  | Do you store water in your house or around your property? | | | | | | | | ☐ No **SKIP TO 35**  ☐ Yes  ☐ Not Sure **SKIP TO 35**  ☐ *Declined* **SKIP TO 35** | | | | | | | |
|  | What do you store water for? | | | | | | | | ☐ Consumption  ☐ Watering plants  ☐ Wash car  ☐ It just happens  ☐ Other ____________________  ☐ NA | | | | | | | |
|  | Do you have air conditioning in your home? | | | | | | | | ☐ No **SKIP TO 38**  ☐ Yes  ☐ *Declined* | | | | | | | |
|  | What type of air-conditioning do you have? | | | | | | | | ☐ Central system  ☐ Window mounted  ☐ Other ________________________ | | | | | | | |
|  | Is there anything that keeps you from using your AC?  ***(mark ALL that apply, DO NOT read)*** | | | | | | | | ☐ Cost of electricity  ☐ Needs repair  ☐ Don’t like AC  ☐ No  ☐ Other ____________________  ☐ NA | | | | | | | |
|  | Do you ever leave your windows open for ventilation?  ***(DO NOT read)*** | | | | | | | | ☐ Never  ☐ Rarely  ☐ Sometimes  ☐ Often  ☐ Always  ☐ NA | | | | | | | |
|  | Do you ever leave your door open for ventilation?  ***(DO NOT read)*** | | | | | | | | ☐ Never  ☐ Rarely  ☐ Sometimes  ☐ Often  ☐ Always  ☐ NA | | | | | | | |
|  | Last week, how many days did you spend an hour or more outdoors doing things like sitting, gardening, working or playing in your yard?  ***(Read options.)*** | | | | | | | | ☐ Never  ☐ Fewer than 3 but at least one a day  ☐ At least 3 days a week  ☐ Most days  ☐ NA | | | | | | | |
|  | Do you ever limit your outdoor activity because you are bothered by mosquitoes? | | | | | | | | ☐ Never  ☐ Rarely  ☐ Sometimes  ☐ Often  ☐ Always  ☐ NA | | | | | | | |
|  | When did a local agency conduct mosquito control last at your house or neighborhood?  ***(Read options.)*** | | | | | | | | ☐ Never  ☐ Within the last year  ☐ Within the last six months  ☐ Within the last month  ☐ Last week  ☐ Not sure  ☐ NA | | | | | | | |
|  | Which of the following methods do you use to avoid being bitten by mosquitoes?  ***(Read options; check all that apply*** | | | | | ☐ Wearing long sleeves  ☐ Burning citronella candles  ☐ Draining stagnant water  ☐ Calling mosquito control  ☐ Burning coils | | | | | | | | | | ☐ Burning “tiki torches”  ☐ Spraying insecticide  ☐ Staying indoors  ☐ Using repellant  ☐ None  ☐ Other _________________ |
|  | Do you have fruit in your home that is kept in the open (not stored in fridge or cabinet)? | | | | | | | | ☐ No **SKIP TO 46**  ☐ Yes  ☐ *Declined* | | | | | | | |
|  | What type of fruit? | | Write everything the interviewer says:  _____________________________________________________ | | | | | | | | | | | | | |
| **Now I’m going to ask you a few questions about the AGO traps we set in your neighborhood** | | | | | | | | | | | | | | | | |
|  | Suppose that there was a proposal to expand the AGO trap mosquito control in your neighborhood. Each home would be given 3 AGO traps to be kept in their yard for a year.  Would you support such a project? | | | | | | | | | | | | | ☐ No **SKIP TO 52**  ☐ Yes  ☐ *Declined* **SKIP TO 52**  ☐ Not sure **SKIP TO 52** | | |
|  | Suppose that all of the traps were provided to households for free, but households would be required to maintain the traps by checking them and replacing the water, hay and sticky board every 2 months. Would you/your household be willing to participate in this program? | | | | | | | | | | | | | ☐ No **SKIP TO 52**  ☐ Yes  ☐ *Declined* **SKIP TO 52**  ☐ Not sure **SKIP TO 52** | | |
|  | How many of the households in your neighborhood do you think would be willing to participate in the program under these conditions? | | | | | | | | | | | | | ☐ None (0%)  ☐ A few (25%)  ☐ About half (50%)  ☐ Many (75%)  ☐ All (100%)  ☐ Don’t know  ☐ *Not sure* | | |
|  | Now suppose that the traps were not given to households for free, but were sold for $15 each ($45 for 3 traps). Households would still be required to maintain the traps as well (checking and changing water every 2 months). Under these conditions, would you/your household be willing to participate in this program? | | | | | | | | | | | | | ☐ No **SKIP TO 52**  ☐ Yes  ☐ *Declined* **SKIP TO 52**  ☐ Not sure **SKIP TO 52** | | |
|  | Under these conditions, how many of the households in your neighborhood do you think would be willing to participate in the program? | | | | | | | | | | | | | ☐ None (0%)  ☐ A few (25%)  ☐ About half (50%)  ☐ Many (75%)  ☐ All (100%)  ☐ Don’t know  ☐ *Not sure* | | |
|  | Please explain your answers. | Why do you think you or others in this area would or would not support an expanded AGO trap program under these different conditions?  _______________________________________________________________________________________________________________________________________________________________________________________________________________ | | | | | | | | | | | | | | |
|  | If you could change something from our trap project what would it be? | | Write everything the interviewer says:  ____________________________________________________________________________________________________________________ | | | | | | | | | | | | | |
|  | We would like to know if you ever had any type of discomfort from having the trap and if so what was it? | | | | | | | Write everything the interviewer says:  ______________________________________________________________________________________________ | | | | | | | | |
|  | Has any of your neighbors asked you about the traps set in your property? | | | | | | | | ☐ No **SKIP TO 56**  ☐ Yes  ☐ *Declined* | | | | | | | |
|  | What have you told them about the trap? | | Write everything the interviewer says:  _____________________________________________________ | | | | | | | | | | | | | |
| **STOP!** | | | | | | | | | | | | | | | | |
|  | Do you think this type of trap worked for reducing mosquito populations in your property, on a scale of 1 to 5 with 1 not working and 5 worked extremely well?  **(READ OPTIONS)** | | | | | | | | | | | ☐ Did not work  ☐ Somewhat worked  ☐ Worked  ☐ Worked well  ☐ Worked extremely well  ☐ NA | | | | |
|  | In reference to the year before we placed the AGO traps in your property, do you think mosquito populations INSIDE your house have:  **(READ OPTIONS)** | | | | | | | | | | | ☐ Increased  ☐ Reduced  ☐ Stayed the same  ☐ NA | | | | |
|  | In reference to the year before we placed the AGO traps in your property, do you think mosquito populations OUTSIDE your house have:  **(READ OPTIONS)** | | | | | | | | | | | ☐ Increased  ☐ Reduced  ☐ Stayed the same  ☐ NA | | | | |
| **END of Intervention groups only!** | | | | | | | | | | | | | | | | |
| **Demographics: Finally, we want to ask a little about you and the people who live in your household.** | | | | | | | | | | | | | | | | |
|  | Do you own this home or do you rent? | | | | | | | | | | | ☐ Own  ☐ Rent  ☐ *Declined* | | | | |
|  | How many rooms does your house have | | | | | | | | | | | ______________ | | | | |
|  | Do you have any of these buildings around your house or near your property? | | | ☐Storage room  ☐Corral  ☐Stable  ☐Chicken coop | | | | | | | | | ☐Pigeon coop  ☐Pigsty  ☐Other ____________________  ☐ *Declined* | | | |
|  | How many people age 18 and over live in this home?  (By “live” I mean sleep at this house this week and not a visitor) | | | | | | | | | | | _______ people  ☐ *Declined* | | | | |
|  | How many children under the age of 5 live in the home? | | | | | | | | | | | _______ people  ☐ *Declined* | | | | |
|  | How many children between the ages of 5 and 18 live in the home? | | | | | | | | | | | _______ people  ☐ *Declined* | | | | |
|  | How many pregnant women have lived in the past year in your house?  **(Stayed more than a week)** | | | | | | | | | | | ___________ Pregnant women | | | | |
|  | Please specify the ethnicity/race you identify with  ***(Check one)*** | | | | | | | | | | | ☐American Indian/Alaskan Native  ☐Asian/Pacific-Islander  ☐Black/African American  ☐White  ☐Multi-racial  ☐Other ______________________  ☐ *Declined* | | | | |
|  | Do you consider yourself Hispanic or Latino? | | | | | | | | | | | ☐Hispanic  ☐Latino | | | | |
|  | What do you consider your primary language?  ***(Mark only one)*** | | | | ☐English  ☐Spanish  ☐Creole | | | | | | | | | | ☐Other ______________  ☐*Decline* | |
|  | What was your total household income from all sources last year?  **(READ ranges)** | | | | | | | | | | | ☐Less than $25,000  ☐$25,000-$49,999  ☐$50,000-$74,999  ☐$75,000-$99,999  ☐$100,000 or more  ☐ *Declined* | | | | |
|  | What is the highest level of education you have completed? | | | ☐No formal education  ☐Less than 9^th^ grade but some formal education  ☐9^th^ to 12^th^  ☐High school graduate/GED | | | | | | | | | ☐Some college, no degree  ☐Associate’s degree  ☐Bachelor’s degree  ☐Graduate or professional degree  ☐ *Declined* | | | |
|  | Have you ever participated in a project to control mosquitoes apart from this one? | | | | | | | | | | | | | ☐ No  ☐ Yes  ☐ *Declined* | | |
|  | If **YES** | | Write everything the interviewer says:  Which?_____________________ Institution______________________ | | | | | | | | | | | | | |
| **Is there anything else you’d like us to know?** | | | | | | | | | | | | | | | | |
| **Thank you very much for your time and valuable information. We appreciate your help!**  *Remember to record finish time.* | | | | | | | | | | | | | | | | |

**Surveys-Housing quality**

| **Lot characteristics (Questions 77 to 79 are done using GIS)** | | | | | | | | | | | | | | |
| --- | --- | --- | --- | --- | --- | --- | --- | --- | --- | --- | --- | --- | --- | --- |
|  | Size of lot (m^2^) | | | | | ☐Depth: _________  ☐Width: _________ | | | | | | ☐Area: _________ | | |
|  | Position of lot | | | | | ☐Corner  ☐Middle | | | | | | ☐End | | |
|  | Lot surface & % of area (m^2^) | | | | | ☐Pave: _______ %  ☐Dirt: ________ %  ☐Grass: _______ % | | | | | | | | |
|  | How much of the yard is vegetated (including grass, flowers, trees, shrubs) | | | | | | | | | | ☐<25 % ☐25-50 %  ☐51-75 % ☐>75 % | | | |
|  | Plants in lot | | | | | ☐thick brush  ☐fruiting plants | | | | | | ☐flowering plants  ☐Other __________________ | | |
|  | Debris on lot | | | | | ☐rocks, bricks  ☐trash  ☐organic material | | | | | | ☐lumber  ☐firewood  ☐Other: ______________ | | |
|  | How long is the vegetation in the yard (grasses, weeds etc.): | | | | | | | | | | ☐Short (<5cm) ☐Medium (5-20 cm)  ☐Long (>20cm) | | | |
|  | Which best describes the tree/ shade cover in the yard? | | | | 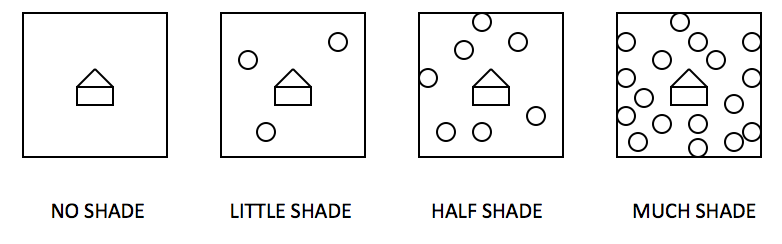  ☐ 1 ☐2 ☐3 ☐4 | | | | | | | | | |
|  | On a scale of 1 to 5 with 1 being extremely orderly/kempt and 5 being an very disorderly, how would you rate this yard? | | | | | | ☐ Extremely orderly (well kempt grass and vegetation, clean well-maintained yard)  ☐ Orderly  ☐ About average  ☐ Disorderly  ☐ Extremely disorderly (uncut grass, stuff piled in the back yard without order, trash etc.) | | | | | | | |
|  | Additional comments | | __________________________________________________________________________________________________________________________________________________________________________________________ | | | | | | | | | | | |
| **House characteristic** | | | | | | | | | | | | | | |
|  | Type of core unit | | | | | ☐ RV  ☐ Mobile home  ☐ Self-help | | | | | | ☐ Custom built  ☐ Manufatured house  ☐ Other: ________________ | | |
|  | Roof shape | | | | | ☐ Pitched  ☐ Flat  ☐ Other: __________________ | | | | | |  | | |
|  | Roof material | | | | | ☐ Cement  ☐ Felt  ☐ Asbestos | | | | | | ☐ Zinc  ☐ Timber Shingles  ☐ Other: ________________ | | |
|  | Does the house have roof/rain gutters? | | | | | | | ☐Yes ☐No | | | |  | | |
|  | Wall material | | | | | ☐ Brick  ☐ Cement block  ☐ Timber frame | | | | | | ☐ Metal frame  ☐ Adobe  ☐ Other: ________________ | | |
|  | No. of rooms | | | Bedrooms/night _________________ | | | | | | Living rooms/day _____________________ | | | | Buffer room between exterior & living spaces ___________________ |
| **Water-holding containers in lot OUTSIDE house** | | | | | | | | | | | | | | |
|  | Rainwater barrels | ☐Yes ☐No | | | | | | | With water: No water:   \|  \|  \|  \| \| --- \| --- \| --- \| | | | | With larvae: With pupae:   \|  \|  \|  \| \| --- \| --- \| --- \| | |
|  | Outdoor pool | ☐Yes ☐No | | | | | | | With water: No water:   \|  \|  \|  \| \| --- \| --- \| --- \| | | | | With larvae: With pupae:   \|  \|  \|  \| \| --- \| --- \| --- \| | |
|  | Outdoor baby pools | ☐Yes ☐No | | | | | | | With water: No water:   \|  \|  \|  \| \| --- \| --- \| --- \| | | | | With larvae: With pupae:   \|  \|  \|  \| \| --- \| --- \| --- \| | |
|  | Plant pots | ☐Yes ☐No | | | | | | | With water: No water:   \|  \|  \|  \| \| --- \| --- \| --- \| | | | | With larvae: With pupae:   \|  \|  \|  \| \| --- \| --- \| --- \| | |
|  | Tires old/new | ☐Yes ☐No | | | | | | | With water: No water:   \|  \|  \|  \| \| --- \| --- \| --- \| | | | | With larvae: With pupae:   \|  \|  \|  \| \| --- \| --- \| --- \| | |
|  | Trash: plastic bags, jugs, tin cans | ☐Yes ☐No | | | | | | | With water: No water:   \|  \|  \|  \| \| --- \| --- \| --- \| | | | | With larvae: With pupae:   \|  \|  \|  \| \| --- \| --- \| --- \| | |
|  | Wheelbarrows | ☐Yes ☐No | | | | | | | With water: No water:   \|  \|  \|  \| \| --- \| --- \| --- \| | | | | With larvae: With pupae:   \|  \|  \|  \| \| --- \| --- \| --- \| | |
|  | Structure with flat roof | ☐Yes ☐No | | | | | | | With water: No water:   \|  \|  \|  \| \| --- \| --- \| --- \| | | | | With larvae: With pupae:   \|  \|  \|  \| \| --- \| --- \| --- \| | |
|  | Gutters (blocked) | ☐Yes ☐No | | | | | | | With water: No water:   \|  \|  \|  \| \| --- \| --- \| --- \| | | | | With larvae: With pupae:   \|  \|  \|  \| \| --- \| --- \| --- \| | |
|  | Roof eaves | ☐Yes ☐No | | | | | | | With water: No water:   \|  \|  \|  \| \| --- \| --- \| --- \| | | | | With larvae: With pupae:   \|  \|  \|  \| \| --- \| --- \| --- \| | |
|  | Low depression areas in lawns | ☐Yes ☐No | | | | | | | With water: No water:   \|  \|  \|  \| \| --- \| --- \| --- \| | | | | With larvae: With pupae:   \|  \|  \|  \| \| --- \| --- \| --- \| | |
|  | A/C trays | ☐Yes ☐No | | | | | | | With water: No water:   \|  \|  \|  \| \| --- \| --- \| --- \| | | | | With larvae: With pupae:   \|  \|  \|  \| \| --- \| --- \| --- \| | |
|  | Animal water bowls | ☐Yes ☐No | | | | | | | With water: No water:   \|  \|  \|  \| \| --- \| --- \| --- \| | | | | With larvae: With pupae:   \|  \|  \|  \| \| --- \| --- \| --- \| | |
|  | Other: | ☐Yes ☐No | | | | | | | With water: No water:   \|  \|  \|  \| \| --- \| --- \| --- \| | | | | With larvae: With pupae:   \|  \|  \|  \| \| --- \| --- \| --- \| | |
|  | Other | ☐Yes ☐No | | | | | | | With water: No water:   \|  \|  \|  \| \| --- \| --- \| --- \| | | | | With larvae: With pupae:   \|  \|  \|  \| \| --- \| --- \| --- \| | |
| **Water-holding containers in lot INSIDE house** | | | | | | | | | | | | | | |
|  | Plant pots | ☐Yes ☐No | | | | | | | With water: No water:   \|  \|  \|  \| \| --- \| --- \| --- \| | | | | With larvae: With pupae:   \|  \|  \|  \| \| --- \| --- \| --- \| | |
|  | Pet water bowls | ☐Yes ☐No | | | | | | | With water: No water:   \|  \|  \|  \| \| --- \| --- \| --- \| | | | | With larvae: With pupae:   \|  \|  \|  \| \| --- \| --- \| --- \| | |
|  | Other | ☐Yes ☐No | | | | | | | With water: No water:   \|  \|  \|  \| \| --- \| --- \| --- \| | | | | With larvae: With pupae:   \|  \|  \|  \| \| --- \| --- \| --- \| | |
|  | Other | ☐Yes ☐No | | | | | | | With water: No water:   \|  \|  \|  \| \| --- \| --- \| --- \| | | | | With larvae: With pupae:   \|  \|  \|  \| \| --- \| --- \| --- \| | |
