# Supplementary material for: The Eco-Bio-Social Factors That Modulate Aedes aegypti Abundance in South Texas Border Communities
Source: Insects. 2021 Feb 21;12(2):183. doi: 10.3390/insects12020183 (PMC7926310; doi:10.3390/insects12020183)
Supplement: Supplementary file 1 [file insects-12-00183-s001.zip › Supplemental/Materials S2-Statistical Analysis.docx]

**Materials S2:**

**Statistical Analysis**

**Table S1.** Dimension reduction methods (DRM) used for the KAP and housing surveys of communities from the LRGV, Texas.

| **Survey** | **DRM** | **Indices** | **Categorical variables** | **Continuous variables** |
| --- | --- | --- | --- | --- |
| **KAP** | MCA | AP1 | Stores water in property; open windows for ventilation; open door for ventilation; unrefrigerated fruit in the house, education, income |  |
|  | PCA | AP2 |  | No. of rooms in house; No. of additional premises in peridomicile, Lot size; total children ≤ 5 years, total children 6 to 17 years |
| **House quality** | FAMD | Yard | Vegetation coverage; messy yard; length of grass; % shaded; core unit | debris on lot; tires; other containers |
|  | PCA | Window |  | Window total; glazing quality; exterior screen total; exterior screen openable; exterior screen seal holes; exterior screen holes; window AC units; AC seal holes |
|  | PCA | Door |  | Total doors, total exterior doors, doors with screen, door screen with holes, gap under door, threshold covers, door brush/edge |

MCA: Multiple Component analysis; PCA: Principal Component analysis; FAMD: Factor Analysis of Mixed Data

**Table S2**. Household demographics

| **Question** | **Variable** | **No. positive responses/total (%)** |
| --- | --- | --- |
| Gender of Interviewee | Female | 22/39 (56.4) |
|  | Male | 15/39 (38.5) |
|  | Couple | 2/39 (5.1) |
| Lived in the LRGV | 1-5 years | 2/39 (5.1) |
|  | > 5 years | 37/39 (94.9) |
| Traveled outside the US in the last year | Yes | 15/39 (38.5) |
|  | Endemic area for arboviral disease | 13/15 (86.7) |
| Children | Pregnant women in the house | 3/39 (7.8) |
|  | ≤ 5 years of age | 12/39 (30.8) |
|  | 6 – 17 years of age | 17/39 (43.6) |
| Total people living in the home | 1 ­– 3 people | 17/39 (43.6) |
|  | 4 – 6 people | 17/39 (43.6) |
|  | 7 – 9 people | 5/39 (12.8) |
| Ethnicity | Hispanic or Latin descent | 38/39 (97.4) |
| Language | Spanish as first form of communication | 31/39 (79.5) |
| Household income | < $24,999 | 22/35 (62.9) |
|  | $25,000­ – $74,999 | 6/35 (17.1) |
|  | > $75,000 | 7/35 (20) |
| Education | No formal education | 1/39 (2.6) |
|  | < 9^th^ with some formal education | 14/39 (35.9) |
|  | 9^th^ to 12^th^ | 4/39 (10.3) |
|  | High school graduate/GED | 8/39 (20.5) |
|  | Some college | 3/39 (7.7) |
|  | Associate’s degree | 1/39 (2.6) |
|  | Bachelor’s degree | 4/39 (10.3) |
|  | Graduate or professional degree | 4/39 (10.3) |

The MCA analysis for the AP1 variables showed that the first dimension accounted for 17.4% of the overall variation and that the second dimension accounted for 13.9% (Figure S1A). We observed a strong correlation between the variables open windows for ventilation (eta^2^ = 0.517) and open doors for ventilation (eta^2^ = 0.438) (Figure S1B). We identified low correlation effects for the other variables and low contribution of almost all variables to both dimensions of AP1. The PCA analysis for the AP2 variable analysis showed that 30.4% and 24.8% of the variation was accounted in the first and second PC, respectively. The AP2.1 loading was for children ≤ 5 years (-0.80): this index can be viewed as a measure of the lack of children ≤ 5 years of age in a house. If the index increases fewer number of children ≤ 5 years will be found in the house. The AP2.2 loadings were for children 6 to 17 years (0.79) and lot size (0.72): this index can be viewed as a measure of property size and the quantity of children 6 to 17 years of age (Table S3). If one of the variables increases the other tends to increase as well, suggesting that larger properties will have more children of 6 to 17 years (Figure S1C).

**Table S3**: Loadings of the principal component 1 (PC1) and 2 (PC2) of the AP2 variable analysis, with its corresponding contribution (ctr) and squared cosine (Cos^2^).

| Variable | PC1 | Ctr | Cos^2^ | PC2 | ctr | Cos^2^ |
| --- | --- | --- | --- | --- | --- | --- |
| Rooms | 0.59 | 23.02 | 0.35 | 0.23 | 4.44 | 0.06 |
| Rooms Premise | 0.59 | 22.73 | 0.35 | -0.11 | 0.96 | 0.01 |
| Below 5 | -0.80 | 42.51 | 0.65 | 0.18 | 2.58 | 0.03 |
| Six to 17 | -0.23 | 3.59 | 0.06 | 0.79 | 50.24 | 0.62 |
| Lot size | 0.35 | 8.15 | 0.12 | 0.72 | 41.78 | 0.52 |


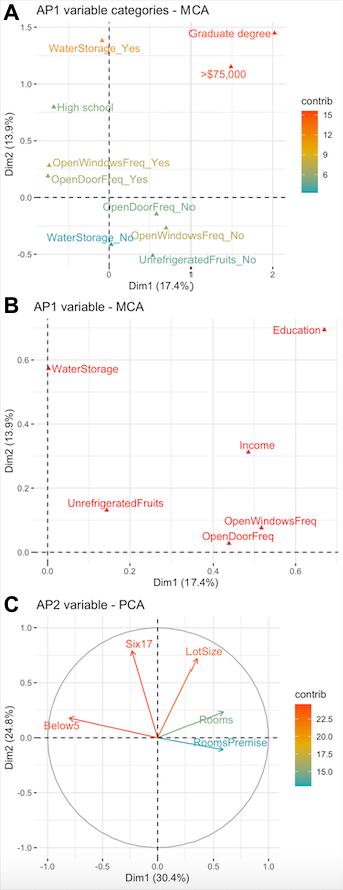


**Figure S1**. Biplots of the Dimension Component 1 (Dim1) and 2 (Dim2) of the Knowledge, Attitude and Practices survey. A) AP1 top ten variable categories with the highest quality of representation of the Multiple Correspondence Analysis (MCA). B) Biplot of the total variables used for the AP1 index. C) Biplot of the Principal Component Analysis (PCA) of the AP2 variables, PC1 and PC2.

The FAMD analysis of the yard variables showed that 20.9% and 14.2% of the variance was accounted in the first and second factors, respectively (Figure S2A). The loadings for the variables messy yard (Disorderly = 2.10), total number of other containers (0.76), and total amount of debris on lot (0.77) were highly correlated and contributed the most to factor 1. The loadings for the variables that contributed the most to the second factor were having an RV core unit (5.96) and no shade (3.83) (Figure S2B-C). 35% of the variability is explained by these two factors.


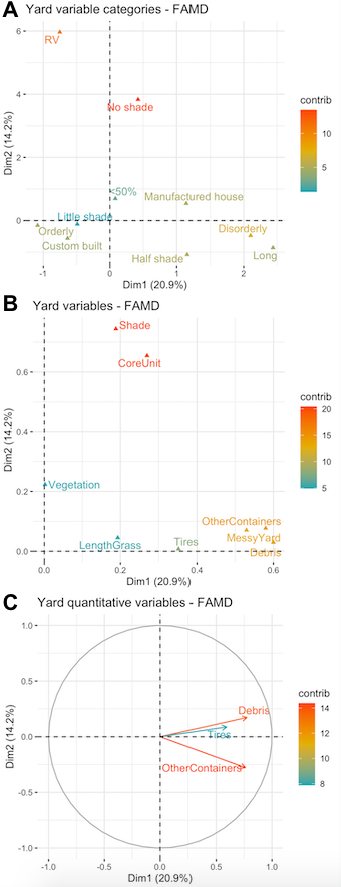


**Figure S2**. Biplots of the Factor Components 1 (Dim1) and 2 (Dim2) of the Yard housing quality survey. A) Yard PC top ten variable categories with highest quality of the representation of the Factor Analysis of Mixed Data (FAMD). B) Biplot between the yard variables and the Dim1–2. C) Biplot between quantitative variables and Dim1–2.

The PCA analysis for the window variables showed that 27.4% and 23.2% of the variation was accounted in the first and second PC, respectively. The Window1 loadings were for exterior screen total (0.87) and windows total (0.65). The Window1 index can be viewed as a measure of quantity of windows. The Window2 loadings were for exterior screen holes (0.86) and exterior screen seal holes (0.87) (Table S4). This index can be viewed as a measurement of the poor quality of screens in windows, with higher values denoting poorer quality. If one of the variables increases the other tends to increase as well (Figure S3A). The PCA analysis of the door variables showed that the first and second PC accounted for 36.7% and 25.7% of the variation, respectively. The Door1 loadings were for doors with threshold covers (-0.84), doors with brushes (-0.79) and door screen with holes (0.60). The Door1 index can be viewed as a measurement of poor quality of doors, it increases with increasing numbers of holes in the screen and doors that do not have threshold covers and door brushes. The Door2 loadings were for doors that have direct access to the exterior (0.85) and total number of doors (0.84) (includes doors that have secondary access to the exterior through the garage) (Table S4). The Door2 index is basically a measurement on the total number of doors with exterior access in a house (Figure S3B).

**Table S4**: Loadings of the Principal Component 1 (PC1) and 2 (PC2) of the Window and Door variable analysis, with its corresponding contribution (ctr) and squared cosine (Cos^2^).

| **Variable** | **PC1** | **ctr** | **Cos^2^** | **PC2** | **ctr** | **Cos^2^** |
| --- | --- | --- | --- | --- | --- | --- |
| **Window:** |  |  |  |  |  |  |
| Window Total | 0.65 | 18.97 | 0.42 | 0.23 | 2.96 | 0.05 |
| Glazing holes | 0.08 | 0.31 | 0.01 | -0.31 | 5.13 | 0.09 |
| Ext. screen total | 0.87 | 34.77 | 0.76 | 0.13 | 0.85 | 0.02 |
| Ext. screen openable | 0.33 | 4.99 | 0.12 | 0.20 | 2.18 | 0.04 |
| Ext. screen seal holes | -0.05 | 0.10 | 0.01 | 0.87 | 40.62 | 0.75 |
| Ext. screen holes | -0.17 | 1.27 | 0.03 | 0.85 | 39.32 | 0.73 |
| Window AC | -0.63 | 18.22 | 0.39 | 0.37 | 7.40 | 0.14 |
| Window AC holes | -0.69 | 21.37 | 0.47 | -0.17 | 1.53 | 0.03 |
| **Door:** |  |  |  |  |  |  |
| Door Total | 0.09 | 0.31 | 0.01 | 0.84 | 34.53 | 0.71 |
| Ext. Doors | 0.18 | 1.12 | 0.03 | 0.86 | 35.52 | 0.73 |
| Door screen | 0.76 | 19.86 | 0.58 | 0.40 | 7.76 | 0.16 |
| Door seal holes | 0.59 | 12.28 | 0.36 | -0.28 | 2.49 | 0.05 |
| Door screen holes | 0.66 | 15.20 | 0.44 | 0.32 | 4.89 | 0.10 |
| Holes under door | 0.40 | 5.53 | 0.16 | -0.38 | 7.09 | 0.15 |
| Threshold covers | -0.84 | 24.18 | 0.70 | 0.29 | 3.97 | 0.08 |
| Door brush | -0.79 | 21.50 | 0.63 | 0.28 | 3.75 | 0.07 |


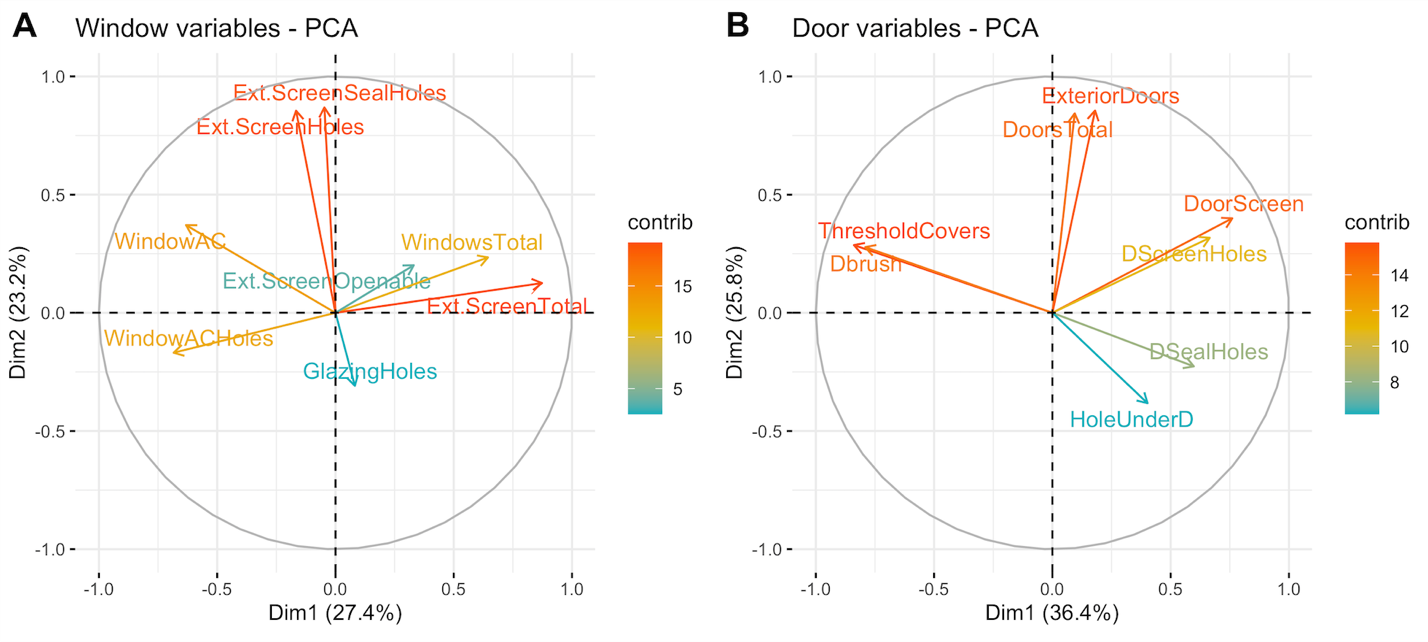


Supplemental Figure 3. Biplots of the Principal Component 1 (Dim1) and 2 (Dim2) of the housing variables for window and door. A) Biplots between the window variables and the Principal Component Analysis (PCA), PC1–2. B) Biplots between the door variables and the PCA, PC1–2.

To evaluate if our best fit model of indoor female *Ae. aegypti* abundance explains significantly more variability than a null model with no predictors, we carried out an ANODE analysis. We observed that our best fit model had a significant difference when compared to the null (Chi^2^= 16.52, df= 8, p= 0.03). This pattern was also observed when comparing the best fit model for outdoor female abundance to a null model (Chi^2^= 23.12, df= 9, p= 0.006). The results show that there is some relationship between our predictors and female abundance in the LRGV.

**Table S5.** Generalized linear model (NB2) parameter selection for the indoor abundance of female *Ae. aegypti* in South Texas.

| **Model** | **Parameters in model** | **AIC** |
| --- | --- | --- |
| 1 | TypeAC + OpenWindow + OpenDoor + WaterStorage + OtherContainers + Income + Outdoor female + AP2.1 + AP2.2 + Window1 + Window2 + Door2 | 145.8 |
| 2 | TypeAC + OpenWindow + OpenDoor + WaterStorage + OtherContainers + Income + Outdoor female + AP2.1 + AP2.2 + Window1 + Door2 | 143.9 |
| 3 | TypeAC + OpenWindow + OpenDoor + WaterStorage + OtherContainers + Income + Outdoor female + AP2.1 + Window1 + Door2 | 142.2 |
| 4 | TypeAC + OpenWindow + OpenDoor + WaterStorage + OtherContainers + Outdoor female + AP2.1 + Window1 + Door2 | 141.1 |
| **5** | **TypeAC + OpenWindow + WaterStorage + OtherContainers + Outdoor female + AP2.1 + Window1 + Door2** | **139.7** |
| 6 | TypeAC + OpenWindow + OtherContainers + Outdoor female + AP2.1 + Window1 + Door2 | 141.2 |

Best fit model is marked with bold. AIC: Akaike Information Criterion

**Table S6**. Generalized linear model (NB2) parameter selection for the outdoor abundance of female *Ae. aegypti* in South Texas.

| **Model** | **Parameters in model** | **AIC** |
| --- | --- | --- |
| 7 | Vegetation + MessyYard + OpenWindow + OpenDoor + WaterStorage + OtherContainers + Tires + Income + AP2.1 + AP2.2 + Window1 + Window2 + Door1 + Door2 | 337.2 |
| 8 | Vegetation + MessyYard + OpenDoor + WaterStorage + OtherContainers + Tires + Income + AP2.1 + AP2.2 + Window1 + Window2 + Door1 + Door2 | 340.9 |
| 9 | Vegetation + MessyYard + OpenDoor + WaterStorage + OtherContainers + Tires + Income + AP2.1 + AP2.2 + Window1 + Door1 + Door2 | 338.9 |
| 10 | Vegetation + OpenDoor + WaterStorage + OtherContainers + Tires + Income + AP2.1 + AP2.2 + Window1 + Door1 + Door2 | 337.2 |
| 11 | Vegetation + OpenDoor + OtherContainers + Tires + Income + AP2.1 + AP2.2 + Window1 + Door1 + Door2 | 335.4 |
| 12 | Vegetation + OpenDoor + Tires + Income + AP2.1 + AP2.2 + Window1 + Door1 + Door2 | 333.6 |
| **13** | **Vegetation + OpenDoor + Tires + Income + AP2.1 + AP2.2 + Door1 + Door2** | **332.9** |

Best fit model is marked with bold. AIC: Akaike Information Criterion
